# Goldilocks and RNA: where Mg^2+^ concentration is just right

**DOI:** 10.1093/nar/gkad124

**Published:** 2023-03-29

**Authors:** Rebecca Guth-Metzler, Ahmad Mohyeldin Mohamed, Elizabeth T Cowan, Ashleigh Henning, Chieri Ito, Moran Frenkel-Pinter, Roger M Wartell, Jennifer B Glass, Loren Dean Williams

**Affiliations:** School of Chemistry and Biochemistry, Georgia Institute of Technology, Atlanta, GA 30332, USA; NASA Center for the Origin of Life, Georgia Institute of Technology, Atlanta, GA 30332, USA; School of Chemistry and Biochemistry, Georgia Institute of Technology, Atlanta, GA 30332, USA; NASA Center for the Origin of Life, Georgia Institute of Technology, Atlanta, GA 30332, USA; NSF/NASA Center for Chemical Evolution, Atlanta, GA 30332, USA; School of Chemistry and Biochemistry, Georgia Institute of Technology, Atlanta, GA 30332, USA; School of Chemistry and Biochemistry, Georgia Institute of Technology, Atlanta, GA 30332, USA; School of Chemistry and Biochemistry, Georgia Institute of Technology, Atlanta, GA 30332, USA; NASA Center for the Origin of Life, Georgia Institute of Technology, Atlanta, GA 30332, USA; Institute of Chemistry, The Hebrew University of Jerusalem, 91904, Israel; NASA Center for the Origin of Life, Georgia Institute of Technology, Atlanta, GA 30332, USA; School of Biological Sciences, Georgia Institute of Technology, Atlanta, GA 30332, USA; Petit Institute of Bioengineering and Bioscience, Georgia Institute of Technology, Atlanta, GA 30332, USA; NASA Center for the Origin of Life, Georgia Institute of Technology, Atlanta, GA 30332, USA; Petit Institute of Bioengineering and Bioscience, Georgia Institute of Technology, Atlanta, GA 30332, USA; School of Earth and Atmospheric Sciences, Georgia Institute of Technology, Atlanta, GA 30332, USA; School of Chemistry and Biochemistry, Georgia Institute of Technology, Atlanta, GA 30332, USA; NASA Center for the Origin of Life, Georgia Institute of Technology, Atlanta, GA 30332, USA; NSF/NASA Center for Chemical Evolution, Atlanta, GA 30332, USA; Petit Institute of Bioengineering and Bioscience, Georgia Institute of Technology, Atlanta, GA 30332, USA

## Abstract

Magnesium, the most abundant divalent cation in cells, catalyzes RNA cleavage but also promotes RNA folding. Because folding can protect RNA from cleavage, we predicted a ‘Goldilocks landscape’, with local maximum in RNA lifetime at Mg^2+^ concentrations required for folding. Here, we use simulation and experiment to discover an innate and sophisticated mechanism of control of RNA lifetime. By simulation we characterized RNA Goldilocks landscapes and their dependence on cleavage and folding parameters. Experiments with yeast tRNA^Phe^ and the *Tetrahymena* ribozyme P4–P6 domain show that structured RNAs can inhabit Goldilocks peaks. The Goldilocks peaks are tunable by differences in folded and unfolded cleavage rate constants, Mg^2+^ binding cooperativity, and Mg^2+^ affinity. Different folding and cleavage parameters produce Goldilocks landscapes with a variety of features. Goldilocks behavior allows ultrafine control of RNA chemical lifetime, whereas non-folding RNAs do not display Goldilocks peaks of protection. In sum, the effects of Mg^2+^ on RNA persistence are expected to be pleomorphic, both protecting and degrading RNA. In evolutionary context, Goldilocks behavior may have been a selectable trait of RNA in an early Earth environment containing Mg^2+^ and other metals.

## Graphical Abstract

**Graphical Abstract:**
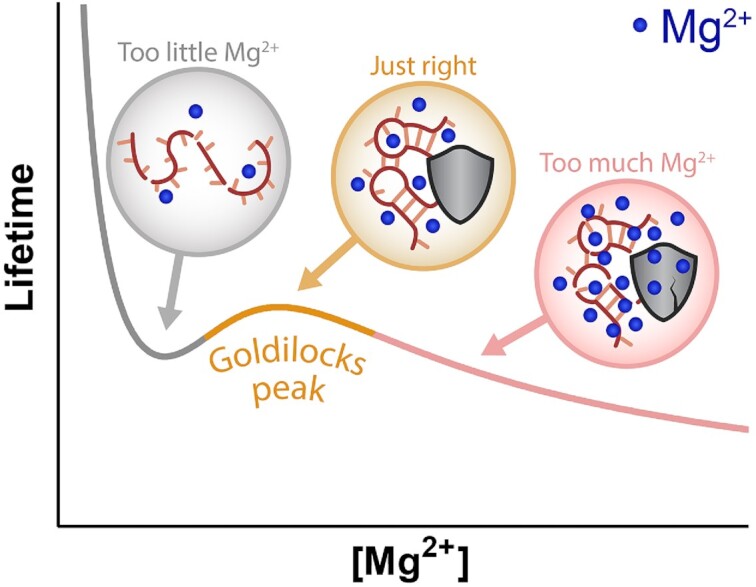
Too little Mg^2+^ accelerates RNA cleavage by minimizing folding. Too much Mg^2+^ accelerates RNA cleavage by over-riding protection. The in-between Goldilocks peak of Mg^2+^ is just right.

## INTRODUCTION

Universal biopolymers (DNA, RNA, protein and polysaccharide) are ephemeral (1,2). Biopolymers hydrolyze spontaneously in aqueous media, degrading to monomers. In dilute aqueous solution, hydrolysis of biopolymers is always thermodynamically favored (3–5). However, low rates of hydrolysis, due to kinetic trapping, allow biopolymers to persist for extended periods of time (1). RNA is especially labile (6), although rates of RNA hydrolysis are modulated by cations, sequence, folding, temperature and proteins (7–9).

**Figure 1.**
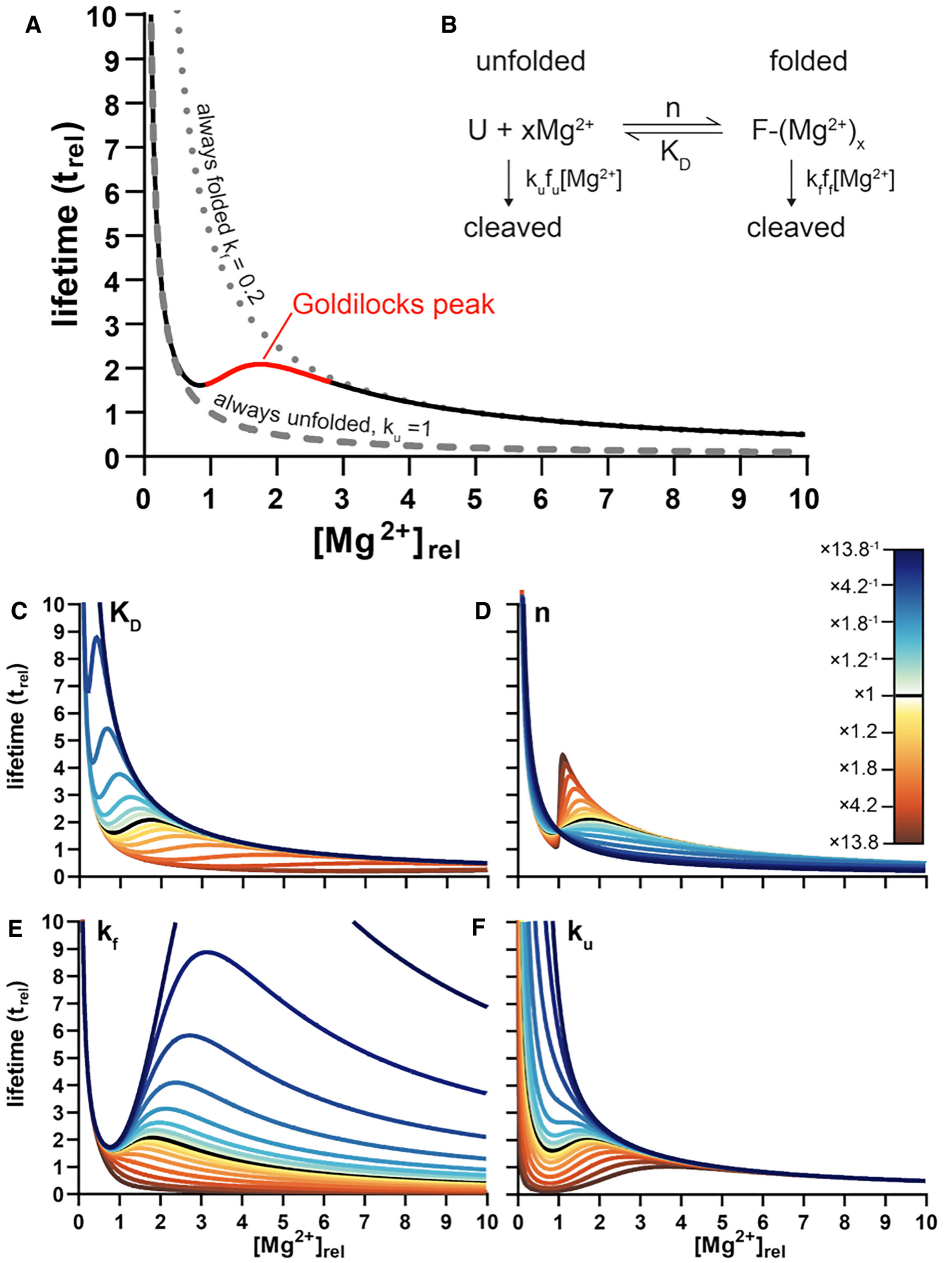
Goldilocks behavior of RNA is predicted by simulations. (**A**) Simulations reveal the influence of [Mg^2+^] on the chemical lifetime of an RNA that is cleaved more slowly in the folded state than in the unfolded state (black/red line). The Goldilocks peak is highlighted in red. The lifetime of an always unfolded RNA is shown by a dashed line (*k*_u_ = 1 *t*_rel_^−1^[Mg^2+^]_rel_^−1^). The lifetime of an always folded RNA is shown by a dotted line (*k*_f_ = 0.2 *t*_rel_^−1^[Mg^2+^]_rel_^−1^). An RNA that shifts between unfolded and folded states shifts between unfolded and folded lifetimes, to establish a Goldilocks peak. Goldilocks behavior requires conversion from unfolded to folded and a lower cleavage constant of folded vs. unfolded RNA (*k*_f_ < *k*_u_). (**B**) A two-state reaction mechanism. U is unfolded RNA and F is folded RNA. (**C–F**) Effects of change of a single parameter while other parameters are held constant. Change of (**C**) *K*_D_, (**D**) *n*, (**E**) *k*_f_ and (**F**) *k*_u_. Each parameter was varied by multiplication or division by 1 + (0.1 × 2^*i*^) (*i* = 1, 2, 3, …, 8). For this representation, [Mg^2+^] was converted to [Mg^2+^]_rel_ where 1 [Mg^2+^]_rel_ = *K*_D_ = 0.022 mM Mg^2+^. Lifetime (*t*) was converted to *t*_rel_ where *t*_rel_ = 1 when [Mg^2+^]_rel_ = 1 and the cleavage constant(s) are fixed at 1 *t*_rel_^−1^[Mg^2+^]_rel_^−1^.

Here we document and characterize Goldilocks behavior of RNAs, with local maxima of chemical lifetimes bounded by conditions of lower lifetimes (Figure 1A). A Goldilocks landscape is a continuum of conditions, some of which are just right for RNA persistence. We predicted Goldilocks landscapes for RNA because Mg^2+^ directly increases RNA cleavage rates by one mechanism and indirectly decreases cleavage rates by a different mechanism. Mg^2+^, the most abundant divalent cation in cells (10), degrades RNA by catalyzing in-line attack of the 2’-oxygen on the backbone phosphate (7,11–16). Mg^2+^ can also protect against degradation, by facilitating RNA folding (17–22). The mechanism of protection involves converting conformationally flexible RNA to more structured RNA (23), which is less likely to adopt the geometry required for cleavage (24). A Goldilocks landscape can arise when a given factor acts as a double-edged sword that differentially degrades and protects. Here, we investigated RNA Goldilocks behavior through simulation and experiment.

In simulations, Goldilocks behavior is observed under a broad variety of parameters that influence RNA folding and cleavage. Goldilocks landscapes are influenced by folding mechanism, Mg^2+^-dependency of folding, and folded and unfolded cleavage rate constants. Goldilocks landscapes are not accessible to RNAs that do not fold or unfold.

In our experiments, Goldilocks behavior is observed for well-established model RNAs, including yeast tRNA^Phe^ (25,26) and the *Tetrahymena* Group I Ribozyme P4–P6 domain (27,28). An experimental comparison of the Goldilocks landscapes of tRNA and P4–P6 RNA indicates that the Goldilocks phenomena is retained even as the landscape is influenced by sequence and chemical modification. In experiments, Goldilocks peaks were observed when RNA is ∼95% folded; a control RNA that does not fold, rU_20_ (polyuridylic acid 20-mer), does not display Goldilocks behavior.

Goldilocks behavior of RNA suggests intrinsic sophistication, allowing ultrafine control of chemical lifetime by a variety of inputs (1,29). RNA chemical lifetimes can be tuned by Mg^2+^-mediated shifts into and out of Goldilocks peaks and by remodeling Goldilocks landscapes via sequence and chemical modification. Goldilocks behavior of RNA is consistent with its selection in a primordial world of stringent and conflicting evolutionary demands.

## MATERIALS AND METHODS

### Simulation of RNA lifetime

We mathematically modeled the effect of [Mg^2+^] on folded and unfolded RNA cleavage rate constants which both contribute to an observed cleavage rate constant *k*_obs_. Lifetime is the reciprocal of *k*_obs_.

For a two-state model, the fraction folded is *f*_f_ and the fraction unfolded is *f*_u_. We used the Hill equation to describe extent of folding (30), although any model that reasonably describes RNA folding can be used (Eq. 1a and 1b):


(1a) $$\begin{equation*}{f_f} = \,\frac{1}{{1 + {{\left( {\frac{{{K_D}}}{{\left[ {M{g^{2 + }}} \right]}}} \right)}^n}}}\end{equation*}$$

(1b) $$\begin{equation*}{f_f} + {f_u} = 1\end{equation*}$$

*K*_D_ is equivalent to [Mg^2+^] at the folding midpoint (21) and *n* is the Hill coefficient, which reflects the cooperativity of folding.

RNA cleavage is a second-order phenomena in which the rate depends on [RNA] and [Mg^2+^] (7,14). The observed pseudo-first-order rate constant (*k*_obs_) is proportional to the second-order rate constant (*k*) and [Mg^2+^] (Eq. 2) (31):


(2) $$\begin{equation*}{k_{obs}} = k\left[ {M{g^{2 + }}} \right]\end{equation*}$$

To model RNA lifetime with a two-state folding model, we used two cleavage rate constants: *k*_f_ for folded RNA and *k*_u_ for unfolded RNA. Folding offers protection from cleavage, and therefore *k*_f_ < *k*_u_. For an RNA that can occupy two states, *k*_obs_ is a convolution of cleavage contributions based on fractional occupancies and rate constants for each state (Eq. 3, which is an extension of Eq. 2 with differential cleavage based on folding):


(3) $$\begin{equation*}{k_{obs}} = {f_f}{k_f}\left[ {M{g^{2 + }}} \right] + {f_u}{k_u}\left[ {M{g^{2 + }}} \right]\end{equation*}$$

RNA lifetime is the reciprocal of the observed cleavage rate constant (*k*_obs_) (Eq. 4) (32):


(4) $$\begin{equation*}lifetime = {\left( {{k_{obs}}} \right)^{ - 1}} = {\left( {\frac{{{k_f}\left[ {M{g^{2 + }}} \right]}}{{1 + {{\left( {\frac{{{K_D}}}{{\left[ {M{g^{2 + }}} \right]}}} \right)}^n}}} + \left[ {1 - \frac{1}{{1 + {{\left( {\frac{{{K_D}}}{{\left[ {M{g^{2 + }}} \right]}}} \right)}^n}}}} \right]{k_u}\left[ {M{g^{2 + }}} \right]} \right)^{ - 1}}\end{equation*}$$

Initial folding parameters, *K*_D_ = 0.022 mM Mg^2+^ and *n* = 4.1, for native yeast tRNA^Phe^ were obtained from previous work (18). Initial values of relative rate constants were set to *k*_u_ = 1 and *k*_f_ = 0.2 *t*_rel_^−1^[Mg^2+^]_rel_^−1^, consistent with changes in RNA cleavage rates upon conversion of single strands to duplex (33,34). The software GraphPad Prism 8 was used for simulations.

To model the contribution of folding intermediates to Goldilocks behavior, we modified the two-state model using the approach of Shelton et al. (18) (Supplementary Eq. 1). The transition from unfolded to intermediate is described by the terms *K*_D1_ and *n*_1_, and the transition from intermediate to fully folded is described by *K*_D2_ and *n*_2_. We initialized the three-state equation with *K*_D1_ at 1 [Mg^2+^]_rel_ and *k*_u_/*k*_f_ at 5. We set both *n*_1_ and *n*_2_ to 4.1 and *K*_D2_ for the second transition to 2 [Mg^2+^]_rel_. *k*_i_ was varied.

Our simulations and analysis of experimental data assume that RNA folding is fast relative to cleavage. We assume that Mg^2+^ binding to RNA is not rate-limiting and that cleavage operates over a fixed folding ensemble. These assumptions are based on experiment and theory. For folding, *k* = 10^4^ to 10^−4^ s^−1^ (35–42). For cleavage *k* = 10^−4^ to 10^−7^ s^−1^ (7,12,14,34). To either cleave or fold RNA, Mg^2+^ must first associate with the RNA. Diffuse association (or diffuse ‘binding’) of Mg^2+^ with RNA has a rate constant of *k* ≈ 10^10^ s^−1^, near the rate of diffusion (43), whereas *k* ≈ 10^5^ s^−1^ for specific binding involving first shell coordination (44,45). Increasing RNA length might attenuate Goldilocks behavior if rate of folding were decreased sufficiently (46) such that folding and cleavage occur on the same timescale.

### RNA preparation

Yeast tRNA^Phe^ was purchased from Sigma-Aldrich (R4018). rU_20_ was purchased from Integrated DNA Technologies. T7-transcribed, stabilized P4–P6 RNA was produced as in Athavale et al. (47). Background Mg^2+^ was removed from the RNAs by dialysis in 180 mM NaCl and 50 mM HEPES buffer pH 7.1 using a 10 kDa MWCO filter.

### Circular dichroism

Extent of folding was quantified by CD spectroscopy. A solution of 10 μM tRNA, 8 μM P4–P6 RNA, or 10 μM rU_20_ in 180 mM NaCl and 50 mM HEPES buffer pH 7.1 was added to a cuvette with a 0.1 cm path length. Spectra were accumulated on a Jasco J-815 spectropolarimeter with scan rate of 200 nm/min, bandwidth of 3 nm, and data pitch of 0.2 nm from 220 to 350 nm at 65°C. The RNAs were titrated with small volumes of concentrated known MgCl_2_ solutions. CD spectra were blank-subtracted and smoothed with a moving average. For yeast tRNA^Phe^ and P4–P6 RNA, fraction folded was plotted using the theta value at the wavelength that maximized the difference between spectra (260 nm for tRNA and 260.6 nm for P4–P6 RNA). Theta values were baseline-corrected for the effects of dilution, evaporation, and cleavage over the course of CD data acquisition. Yeast tRNA^Phe^ showed little cleavage during CD data acquisition. Some P4–P6 RNA cleavage was observed, consistent with the longer CD acquisition time and greater RNA length (Supp. Figure 1).

### In-line cleavage of RNA

We approximated native ionic strength at 180 mM NaCl, which required elevated temperature (65°C) to observe Mg^2+^-dependent folding (48). Solutions of 10 μM yeast tRNA^Phe^, 3.8 μM P4–P6 RNA, or 10 μM rU_20_ in 180 mM NaCl, 50 mM HEPES buffer pH 7.1, and variable MgCl_2_ were incubated at 65°C for 48 hour. Reaction mixtures were separated by electrophoresis on 6% or 7% urea PAGE gels run at 120V for 1 hour and stained with SYBR Green II. Stained gels were digitized with an Azure 6000 or Typhoon FLA 9500 Imaging System. Band intensities were quantified using AzureSpot Analysis software to determine the amount of total intact RNA present.

### Sequencing and fragment analysis of P4–P6 RNA cleavage products

RNAs were analyzed by capillary electrophoresis (SeqStudio, Applied Biosystems) following the manufacturer's protocol. Data were analyzed and peaks were aligned in MATLAB. A final concentration of 10 ng/μL uncleaved ‘fresh’ P4–P6 RNA with 0.4 μM of FAM-labeled reverse transcription primer that binds to the 3’ end (5’-AGCTTGAACTGCATCCATATCAACA-3’, Integrated DNA Technologies), 5 mM DTT, 1× first strand buffer (Invitrogen), 0.5 mM each dNTP, and 2 mM of either ddATP, ddCTP, ddGTP or ddTTP was reverse transcribed by SuperScript III (Invitrogen) to create fragments for sequencing. The P4–P6 RNA cleavage reactions containing 5, 10 and 15 mM Mg^2+^ were reverse transcribed in parallel with omission of the ddNTPs for identification of cleavage sites. 1 μl of each reverse transcription was combined with 1 μl GenefloTM 625 size standard ROX ladder (CHIMERx) and 20 μl HiDi (Applied Biosystems). Samples were resolved on a SeqStudio instrument using the FragAnalysis run module.

## RESULTS

### Simulations reveal Goldilocks behavior

We investigated RNA Goldilocks behavior for RNAs that fold in response to Mg^2+^. The simplest model (Figure 1) allows two states (folded and unfolded) and two cleavage rate constants; *k*_u_ is the cleavage rate constant of an unfolded RNA and *k*_f_ is the cleavage rate constant of a folded RNA. The observed rate constant shifts from *k*_u_ when the RNA is fully unfolded, to a weighted average of *k*_u_ and *k*_f_ when the RNA is partially folded, to *k*_f_ when the RNA is fully folded. This model allows a Goldilocks peak of chemical lifetime if *k*_f_ < *k*_u_. From the top of a Goldilocks peak, RNA lifetime decreases if [Mg^2+^] is either increased or decreased. The folding transition in this model is governed by [Mg^2+^], *K*_D_ (for [Mg^2+^] and *n* (the Hill coefficient).

This simple two-state model predicts a Goldilocks landscape of chemical lifetime over a broad range of folding and cleavage parameters (Figure 1C–F). *K*_D_ modulates the position of the Goldilocks peak on the [Mg^2+^] axis; RNAs that fold at lower [Mg^2+^] show a Goldilocks peak at lower [Mg^2+^]. The Hill coefficient *n* modulates the sharpness of the Goldilocks peak without a substantial change in its position; a larger *n* gives a sharper peak. *k*_u_ modifies the slope of the Goldilocks peak on the low [Mg^2+^] side, and *k*_f_ modifies the slope on the high [Mg^2+^] side. The ratio of *k*_u_ to *k*_f_ modulates the intensity of the peak. Goldilocks peaks are absent for RNAs that (i) do not fold, (ii) are always folded, (iii) do not change cleavage rate constant upon folding, or (iv) transition very gradually between differential cleavage realms with varying [Mg^2+^] (Supplementary Figure 2).

Here we define Goldilocks peak intensity as the ratio of the lifetime at the local maximum to the lifetime at the preceding minimum. Positions of maxima and minima were determined from the simulated lifetime derivative across [Mg^2+^] and solving for [Mg^2+^] where slopes are zero. Returning each of those [Mg^2+^] values back into the original lifetime equation solves for the maximum and minimum used for Goldilocks peak intensity. In experiment, the low and high lifetime datapoints were used directly for the ratio.

### Goldilocks behavior in complex models

**Figure 2.**
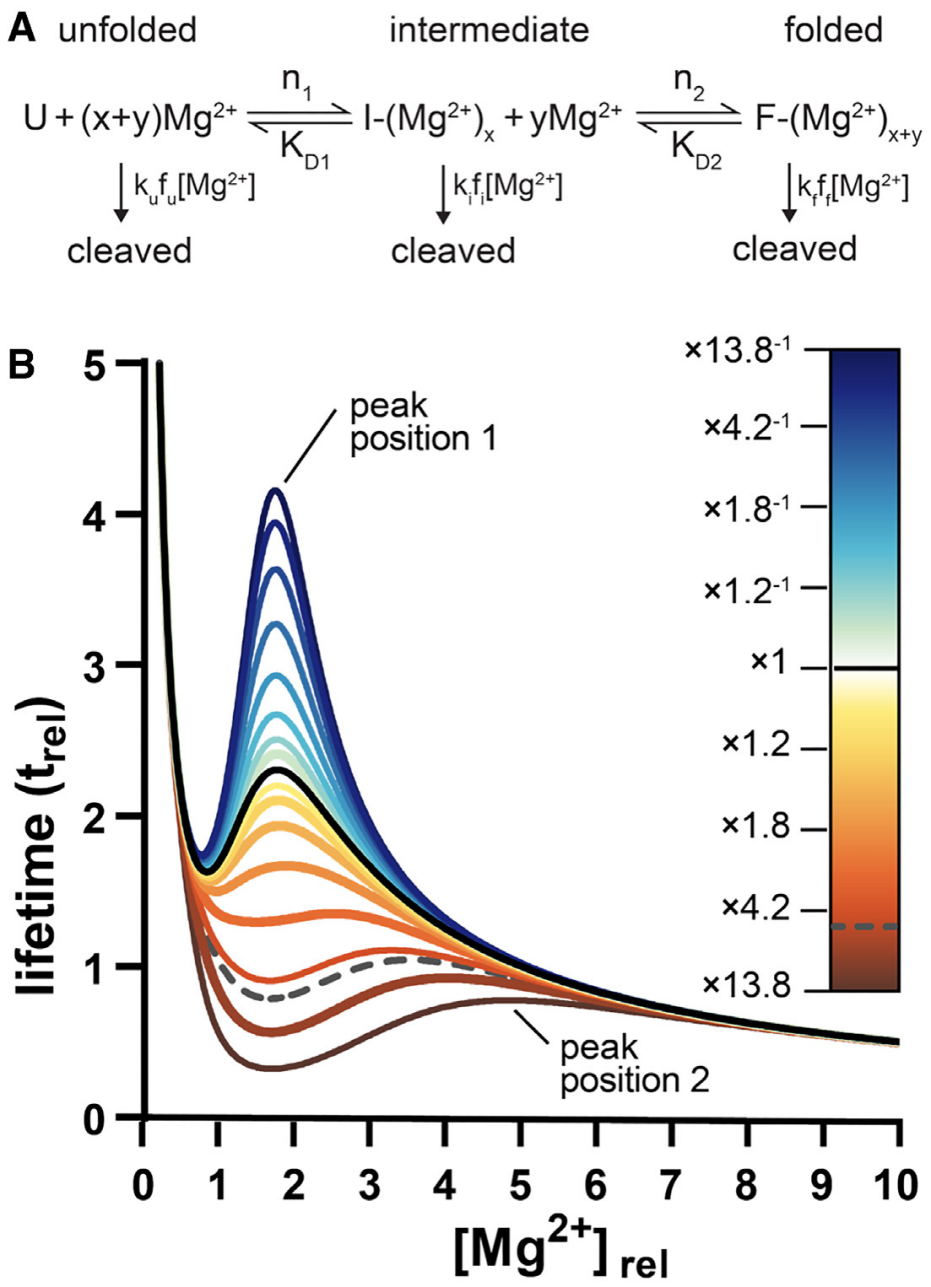
Complex folding models predict Goldilocks peaks. (**A**) In a three-state mechanism, unfolded RNA converts by a first transition to an intermediate and by a second transition to a fully folded state. Unfolded RNA is cleaved with a rate constant *k*_u_, the intermediate is cleaved with a rate constant k_i_, and fully folded RNA is cleaved with a rate constant of *k*_f_. (**B**) In the simulation, *k*_i_ was varied relative to *k*_u_ while other parameters were fixed. The black solid line represents lifetimes when *k*_i_ = *k*_f_. The black dashed line represents the lifetimes when *k*_i_ = *k*_u_. A *k*_i_ < *k*_f_ scenario favors an early (i.e. low [Mg^2+^]) Goldilocks peak at peak position 1 and a *k*_i_ > *k*_u_ scenario favors a late Goldilocks peak at peak position 2.

More realistic RNA folding mechanisms involve intermediate states (18). In a model with intermediates, each intermediate I is associated with specific cleavage rate constant *k*_i_ (Figure 2A). The simulations reveal that the number, intensities, and proximities of Goldilocks peaks depend on the relative magnitudes of the cleavage rate constants and on locations of the folding transitions in [Mg^2+^]-space. For a three-state model with two transitions that are fully resolved in [Mg^2+^]-space, RNA can display two distinct Goldilocks peaks (Figure 2B). When the transitions overlap in Mg^2+^-space, decreasing *k*_i_ tends to increase the intensity of the Goldilocks peak at the [Mg^2+^] where the intermediate population is maximum. Increasing *k*_i_ depresses lifetime at low [Mg^2+^] (peak position 1, Figure 2B) and shifts the Goldilocks peak to higher [Mg^2+^] (peak position 2, Figure 2B). An RNA with a folding intermediate that is cleaved more slowly than the fully folded state (*k*_i_ < *k*_f_) or cleaved more rapidly than the unfolded state (*k*_i_ > *k*_u_) can display especially intense Goldilocks peaks. If an intermediate state has intermediate protection, the net Goldilocks peak is less intense than in the absence of an intermediate.

### Goldilocks intensity

**Figure 3.**
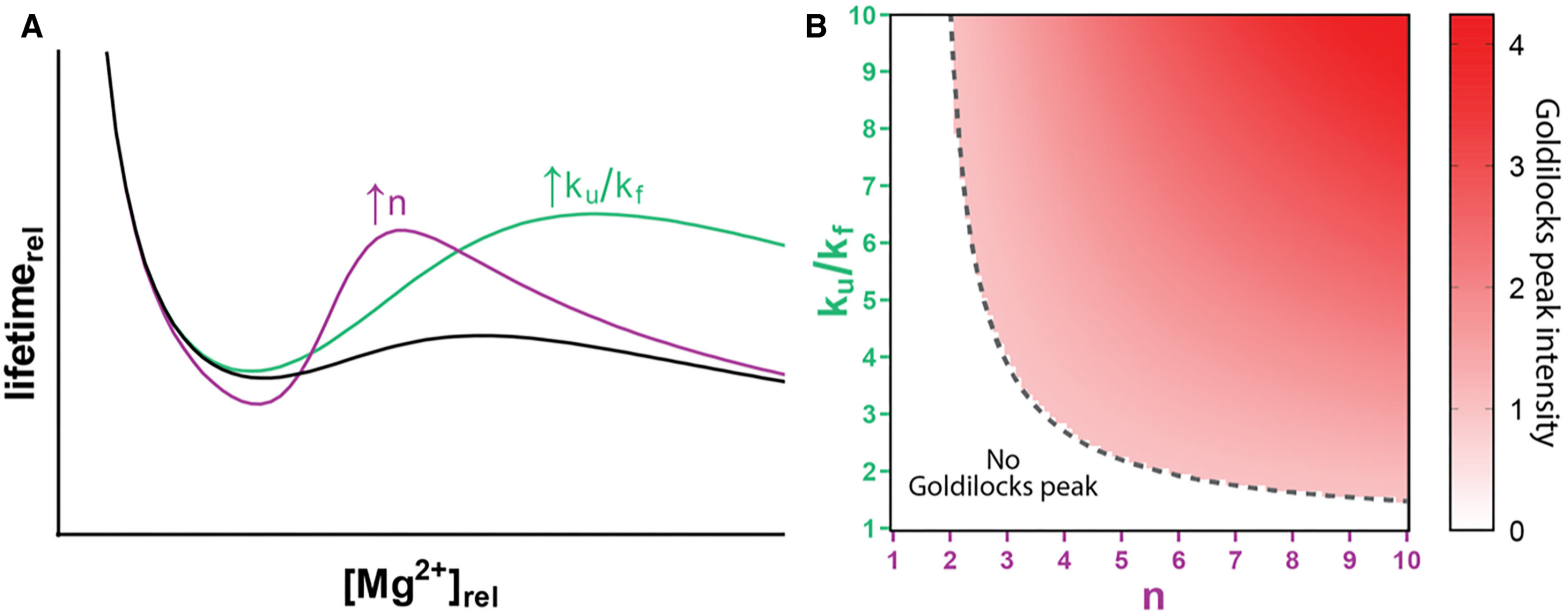
Goldilocks peak intensity increases with *n* and *k*_u_/*k*_f_ ratio. (**A**) A simulated Goldilocks peak (black line) is enhanced by increasing cooperativity of folding (*n*) or increasing the ratio of the unfolded to the folded cleavage rate constants (i.e. increasing relative protection of the folded state). (**B**) Survey of a range of rate constant ratios (*k*_u_/*k*_f_) across a range of *n* values shows that regions with high *k*_u_/*k*_f_, *n*, or both have Goldilocks peaks and regions where these parameters are low do not.

We show that RNA can inhabit a Goldilocks peak of RNA protection flanked by conditions of lability. The level of protection, given by Goldilocks peak intensity, depends on specific RNA properties. We defined Goldilocks peak intensity as the ratio of the peak maximum to minimum, i.e. the ratio of the protected lifetime to the labile lifetime. Using Goldilocks peak intensity, one can compare and rank various RNAs. We observed, in two-state simulations, that Goldilocks peak intensity increases with increased cooperativity of folding (*n*) or with increased extent of protection afforded by folding (decrease of *k*_f_ relative to *k*_u_) (Figure 3A). We surveyed values of *n* and *k*_u_/*k*_f_ to create a Goldilocks intensity map (Figure 3B). A *k*_u_/*k*_f_ = 3 with an *n* = 4 produces a modest Goldilocks peak. Increasing either *n* or *k*_u_/*k*_f_ increases Goldilocks peak intensity. Lowering either *n* or *k*_u_/*k*_f_ disallows a Goldilocks peak unless there is a compensatory increase in the other parameter. We considered area under the curve as an alternative method for quantifying Goldilocks phenomena. By this method, greater area under the curve would correspond to more extensive protection. Intensity and area together describe the shape of a Goldilocks peak. While intensity indicates the protection an RNA achieves near the peak, area can be concentrated locally or widely dispersed in Mg^2+^-space (Supplementary Figure 3). A local description (intensity) appears to be a more useful comparator.

### Experimental observation of a Goldilocks landscape of tRNA

**Figure 4.**
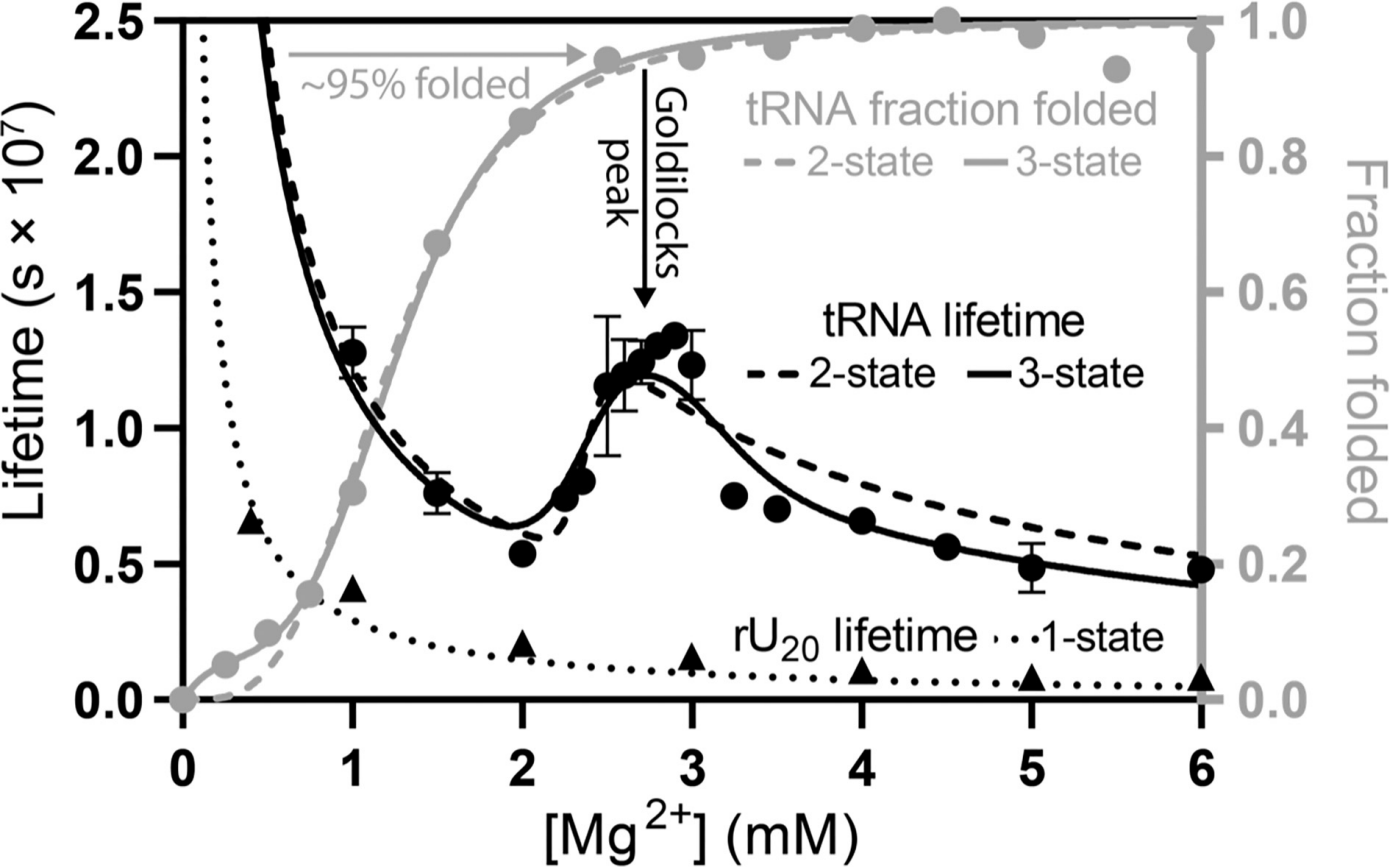
Yeast tRNA^Phe^ shows Goldilocks behavior. Lifetimes of yeast tRNA^Phe^ (black circles) and rU_20_ (black triangles) were determined over a range of [Mg^2+^]. The fraction folded of yeast tRNA^Phe^ (gray circles) was determined by CD. Experimental lifetimes were fit to two-state (black dashed) and three-state (black solid) models. Fraction folded was fit with two-state (gray dashed) and three-state (grey solid) Hill equation models. The three-state model better approximates the lifetime data, with a more intense Goldilocks peak than the two-state model. The tRNA Goldilocks peak is coincident with folding. rU_20_ lifetimes decrease monotonically with no Goldilocks peak (dotted black). rU_20_ did not show a folding transition (Supplementary Figure 6). Lifetimes were determined by quantification of intact RNA resolved by PAGE after 48 hours and normalized per phosphodiester bond. All experiments were conducted in 180 mM NaCl, 50 mM HEPES pH 7.1 at 65°C, with variable [Mg^2+^]. Yeast tRNA^Phe^ lifetime error bars represent the standard deviation of five replicates. Folding and rU_20_ lifetime experiments used one replicate.

To experimentally investigate Goldilocks behavior, we assayed both fraction folded and lifetime of yeast tRNA^Phe^ across a range of [Mg^2+^]. Circular Dichroism showed a clear cooperative folding transition with a [Mg^2+^] midpoint between 1 and 2 mM (Figure 4 and Supplementary Data; Supp. Figure S4). Random coil yeast tRNA^Phe^ folds to the native L-shaped structure upon addition of Mg^2+^ (35,49–54). Chemical lifetime of yeast tRNA^Phe^ showed a distinct Goldilocks peak near 3 mM Mg^2+^, where the tRNA was ∼95% folded (Figure 4 and Supplementary Figure 5). The tRNA lifetime was longer at 3 mM Mg^2+^ than at either 2.0 mM or at 3.5 mM Mg^2+^. In contrast, rU_20_, which does not fold, did not exhibit Goldilocks behavior (Supplementary Figure 6).

We compared the experimental lifetime data with predictions of our models (Figure 4). The observed yeast tRNA^Phe^ Goldilocks landscape is reasonably fit by a two-state model. The fit and observed Goldilocks peaks are centered at the same [Mg^2+^]. The cleavage rate constant of the unfolded tRNA is predicted to be 2.7 times greater than that of the folded tRNA. However, the observed Goldilocks peak is sharper and more intense than predicted by the two-state model. A three-state model provides a better fit to the data, especially in the center of the Goldilocks peak. In the three-state model the cleavage rate constant for the unfolded RNA is predicted to be 3.2 times that of the intermediate and 2.2 times that of the fully folded tRNA (*k*_u_ > *k*_f_ > *k*_i_). The statistical significance of the improved fit of the three-state versus the two-state lifetime and folding models is indicated by residual errors (Supp. Figure 7). Our results are consistent with previous observations of >2 states for folding of yeast tRNA^Phe^ (18,35,49,55). The fundamental conclusion here, which is the prediction and validation of Goldilocks behavior by RNA, is not dependent on the folding model.

Yeast tRNA^Phe^, with an intense Goldilocks peak, appears to fold via a protected intermediate. Prior work has shown that yeast tRNA^Phe^ is most compact in intermediate ionic strength (56–58) suggesting that the folding intermediate is more compact that the native state (however, see reference (59)).

### Experimental observation of a Goldilocks landscape of *Tetrahymena* ribozyme P4–P6 domain RNA

**Figure 5.**
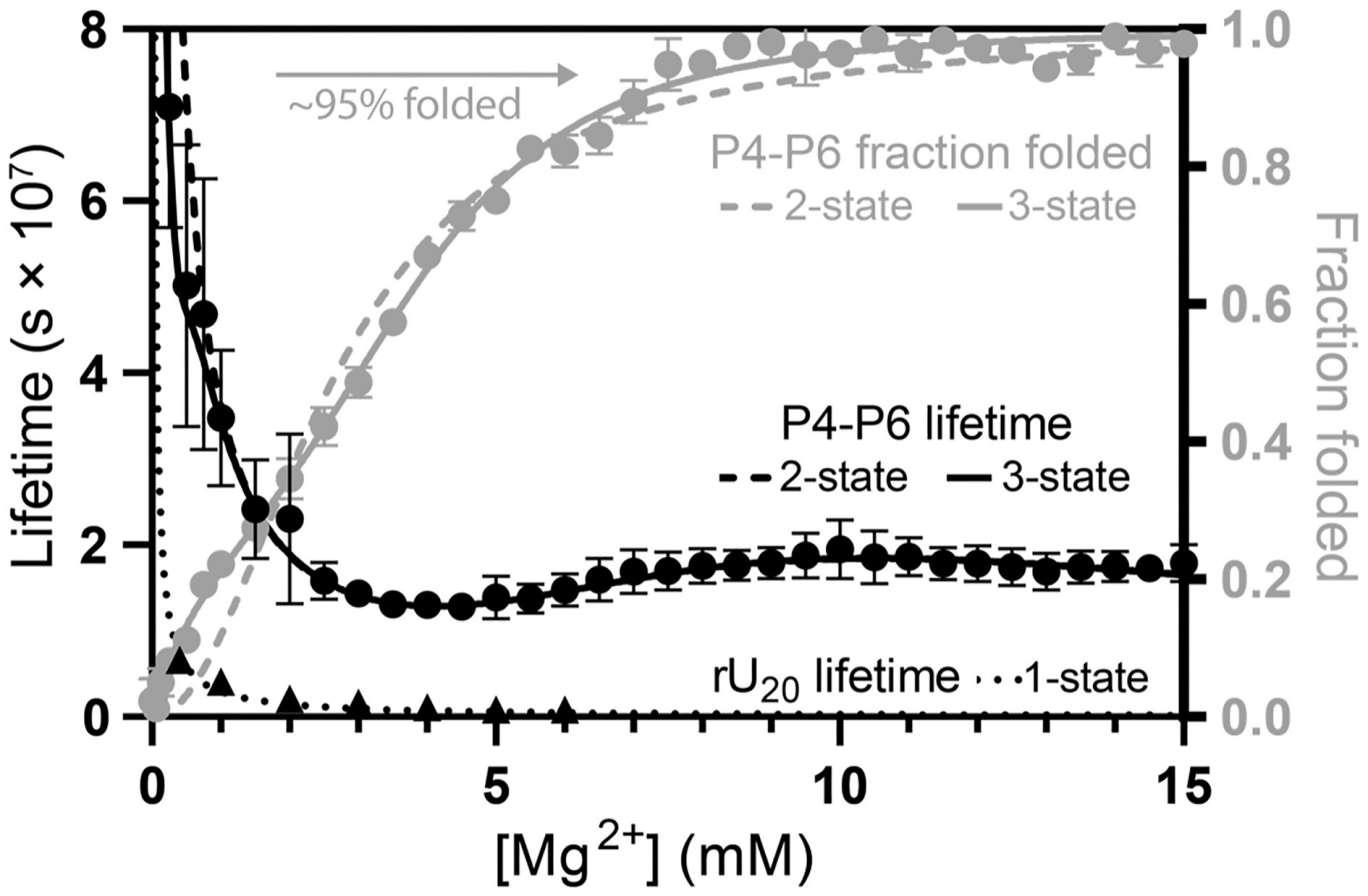
P4–P6 RNA shows Goldilocks behavior. P4–P6 RNA lifetime (black circles) shows a Goldilocks peak when the RNA is near-fully folded. The two-state (dashed line) and three-state (solid line) fits equally capture lifetime within the Goldilocks peak but the three-state fit better approximates lifetime at low [Mg^2+^]. Lifetime is normalized per phosphodiester bond. For a non-folding RNA comparison, rU_20_ lifetime (black triangles) is included and fit with a one state model (dotted line). P4–P6 RNA folding measured by CD (gray circles) is better approximated by a three-state fit (solid gray line) than a two-state fit (dashed gray line). Both folding and lifetime experiments were conducted in 180 mM NaCl, 50 mM HEPES pH 7.1 at 65°C with variable MgCl_2_. Error bars represent standard deviations. P4–P6 RNA lifetimes are averages of four replicates, P4–P6 RNA lifetimes are averages of two replicates. rU_20_ lifetimes were obtained by one replicate.

P4–P6 RNA is a well-established model (27,28) that folds with increasing Mg^2+^ (60,61). The Mg^2+^−dependence of P4–P6 RNA folding by CD (Supplementary Figure 8) and chemical lifetime (Supplementary Figure S9 and Supplementary Data) were determined (Figure 5) by the same methods and under the same conditions as for yeast tRNA^Phe^. P4–P6 RNA has a clear Goldilocks peak that is coincident with RNA folding.

The Goldilocks behavior of P4–P6 RNA is approximated by both the two-state or three-state models. Both models recreate the position in Mg^2+^-space and intensity of the single Goldilocks peak. The peak is produced by the intermediate to folded transition in the three-state model wherein *k*_i_ is 8 times *k*_f_. When constrained to two states, *k*_u_ is 22 times *k*_f_. A low [Mg^2+^] folding transition prior to the transition that forms the Goldilocks peak is approximated by the three-state model. The low [Mg^2+^] trend is captured only by the three-state model. The fit suggests that P4–P6 RNA folds by least three states, even though only two contribute to the Goldilocks peak. This conclusion is supported by residual plots for both lifetime and folding (Supplementary Figure 10) and previous observations that P4–P6 RNA has more than two folding states (60,61).

### Comparison of yeast tRNA^Phe^ and P4–P6 RNA

Both RNAs display Goldilocks peaks in experiment and in simulation. Both RNAs are best fit to models with more than two states. For the tRNA the Goldilocks peak is sharp, with a half-height peak width of around 1 mM Mg^2+^. This level of cooperativity is associated with a pronounced Goldilocks peak. tRNA is noted for its high level of structure (62,63). Conversely, P4-P6 RNA has low cooperativity, which is associated with a broad Goldilocks peak, with a peak width at half-height of around 5 mM Mg^2+^. Goldilocks peak intensity for tRNA is greater (2.4) than for P4–P6 RNA (1.5). The fit parameters of both RNAs are shown in Table 1.

**Table 1.** Fitted parameters for yeast tRNA^Phe^ and P4–P6 RNA

RNA	Exp.^a^	States	*k*_u_ (s^−1^M^−1^)^b^	*k*_i_ (s^−1^M^−1^)^b^	*k*_f_ (s^−1^M^−1^)^b^	*K*_D1_ (mM)	*K*_D2_ (mM)	*n*_1_	*n*_2_
tRNA	lifetime	3	8.7 × 10^−5^	2.7 × 10^−5^	3.9 × 10^−5^	2.2	3.3	15^c^	15^c^
	lifetime	2	8.2 × 10^−5^	–	3.1 × 10^−5^	2.3	–	33.5	–
	folding	3	–	–	–	0.2	1.3	1.1	3.8
	folding	2	–	–	–	–	1.2	–	3.4
P4–P6	lifetime	3	6.1 × 10^−4^	2.8 × 10^−5^	3.5 × 10^−6^	0.5	4.9	3.9	3.4
	lifetime	2	8.7 × 10^−5^	–	3.9 × 10^−6^	–	4.8	–	3.4
	folding	3	–	–	–	2.3	3.8	0.9^d^	2.9
	folding	2	–	–	–	–	2.7	–	2.0

^a^Experiment type, either lifetime analysis by fraction intact in PAGE or folding analysis by CD.

^b^Cleavage rates are per phosphodiester bond.

^c^Values were poorly constrained, the value shown is one that minimizes magnitude while following the data.

^d^ *n* values less than one have literature precedent (64).

### Site specificity of cleavage

**Figure 6.**
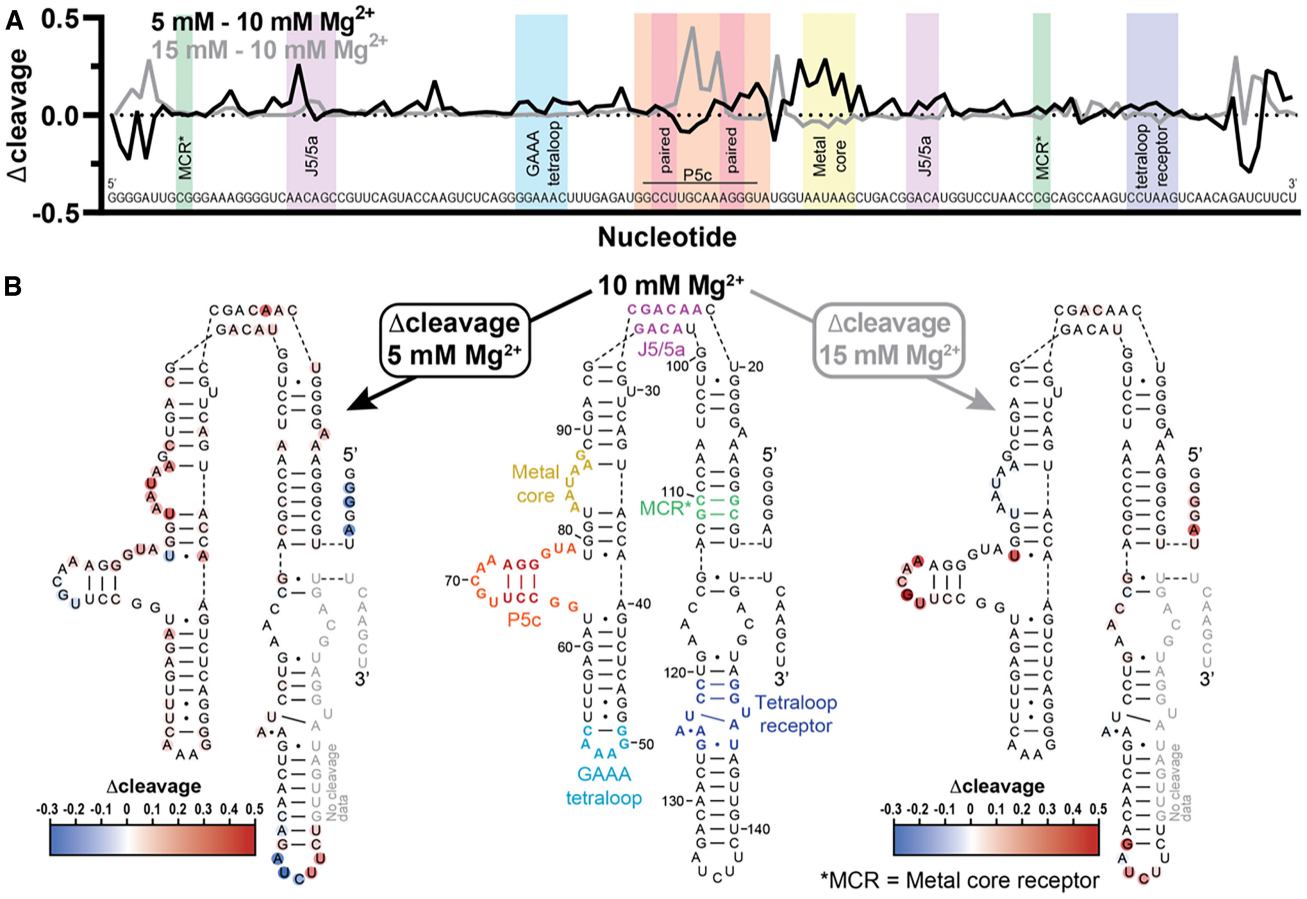
Differences in site specificity of cleavage with changing [Mg^2+^]. (**A**) Δcleavage reports differences in cleavage between P4–P6 RNA at the Goldilocks peak (10 mM Mg^2+^) and at 5 or 15 mM Mg^2+^. Δcleavage indicates highest lifetime at the Goldilocks peak. (**B**) Mapping of cleavage data onto the secondary structure of P4–P6 RNA shows that hot spots for cleavage (red) or protection (blue) relative to P4–P6 RNA at 10 mM Mg^2+^ occur at loops and bulges in the RNA. Important structural features are provided as in Bisaria 2016 (67). Each Δcleavage value is the average of two replicates.

To characterize Goldilocks phenomena at the level of single nucleotide, we quantified cleavage fragments of P4–P6 RNA using a sequencer. We examined the site-specific extent of cleavage under conditions of the Goldilocks peak (10 mM Mg^2+^), on the partially folded shoulder of the peak (5 mM), and on the fully folded should of the peak (15 mM) (Supp. Figure S11 and Supplementary Data). The average extent of cleavage is less under the conditions of the Goldilocks peak (0.18) than in regions flanking the peak (0.21 pre-peak and 0.20 post-peak). Subtracting the 10 mM Mg^2+^ Goldilocks peak condition as a baseline shows nucleotides that experience more cleavage off the peak than on the peak, with larger Δcleavage values indicating greater cleavage (Figure 6A). This information is mapped onto the P4–P6 secondary structure (Figure 6B). The number of detectable cleavage sites and dispersion of cleavage intensities are greatest for the partially folded RNA (at 5 mM Mg^2+^). These sites overwhelm the few nucleotides that are highly cleaved in the folded state (strong negative Δcleavage). In the partially folded realm, double-stranded RNA shows more uniform extent of cleavage than unpaired RNA (65). Variability decreases when the RNA fully folds, where the conformations of essentially all nucleotides become fixed. Here, Δcleavage for 5 mM to 10 mM Mg^2+^ is most variable, especially in bulge and loop-forming regions. When increasing from 10 mM to 15 mM Mg^2+^ a few cleavage hot spots emerge in unpaired regions, where the RNA is most susceptible to cleavage (24).

In P4–P6 RNA, the Mg^2+^-binding metal core does not appear to be folded at 5 mM Mg^2+^, as indicated by extent of cleavage (Figure 6A, B). The metal core folds and is protected at 10 mM Mg^2+^. This region is a representation of the double-edged sword of Mg^2+^; by associating with Mg^2+^ the RNA is protected from Mg^2+^. As expected (66), by its low reactivity relative to other loop or bulge regions, the GAAA tetraloop appears to be fully folded by 5 mM Mg^2+^.

The sequencing data indicate that even though the low resolution P4–P6 RNA PAGE banding patterns remain reasonably constant, relative extent of cleavage at various sites does in fact change. Although the yeast tRNA^Phe^ banding pattern appeared uniform across [Mg^2+^] in gels, we assume significant differences in locations of cleavage upon folding. Site-specific analysis of yeast tRNA^Phe^ was not possible with our method because of base modifications.

## DISCUSSION

By simulation and experiment we validated a Goldilocks model of RNA. Local maxima in lifetime are flanked by conditions of greater lability. RNAs can resist Mg^2+^-mediated cleavage when Mg^2+^ folds the RNA. Increasing [Mg^2+^] beyond the folding threshold increases Mg^2+^-mediated cleavage. We use a Goldilocks framework to explain how lifetime landscapes are modulated by specific characteristics of diverse RNAs. We predict that Goldilocks landscapes are modulated by monovalent cation concentrations, type of divalent cation, RNA sequence and modification, protein and ligand association, and temperature. RNA that does not fold or unfold cannot access Goldilocks protection. Self-cleaving ribozymes are exempt from Goldilocks behavior because their folding increases rates of cleavage.

### Diversity of Goldilocks landscapes

RNA response to [Mg^2+^] is modulated by RNA sequence and chemical modification. The number, intensity, profile, and position in [Mg^2+^]-space of Goldilocks peaks depends on RNA sequence and chemical modification. The position of a Goldilocks peak in [Mg^2+^]-space is determined primarily by the affinity of the RNA for Mg^2+^. A smaller *K*_D_ shifts the Goldilocks peak to lower [Mg^2+^].

Goldilocks behaviors of RNA should extend beyond Mg^2+^ to species such as Fe^2+^, which also promote both RNA folding and cleavage (47,68). Even farther, the general principles of Goldilocks behavior can be applied to any agent that has differential opposing effects on biopolymer lifetime. For example, protein is cleaved by hydrolysis (69). Protein folding decreases rates of hydrolysis (1) and is often promoted by high water activity (70). This model predicts water-defined Goldilocks phenomena for proteins.

### Goldilocks behavior *in vivo*

Our simulations anticipate some RNAs *in vivo* may inhabit Goldilocks peaks. Free Mg^2+^*in vivo* is near 1 mM (71,72) (Table S1). An RNA hairpin ribozyme used as a model is mostly folded at 1 mM Mg^2+^ under molecular crowding, mimicking the cytosol (73). If the minimal [Mg^2+^] required for folding coincides with the *in vivo* [Mg^2+^], RNA may occupy a Goldilocks peak in cells. More specific *in vivo* conclusions remain unresolved thus far because of differences in *in vitro* and *in vivo* conditions and limitations in manipulating *in vivo* [Mg^2+^] (74). Goldilocks landscapes remain to be evaluated *in vivo* and in the context of protein and ligand binding. For mRNAs, with lifetimes *in vivo* of minutes (75–79), spontaneous cleavage might be insignificant. However, long-lived RNAs (tRNAs, ∼9 hours to days (80–83); and rRNAs, ∼5 hours to days (84–88)) might be subject to spontaneous cleavage, governed by the Goldilocks phenomena. Goldilocks behavior could explain in part why cells invest in careful maintenance of Mg^2+^ homeostasis (89). It seems likely that a narrow range of [Mg^2+^] prolongs specific RNA lifetimes *in vivo*.

### Goldilocks and origins of life

RNA can transit between dangerous spaces and safe spaces. Finely controlled metastability, with access to Goldilocks peaks of protection, is most likely an imprint of evolutionary processes (90) during the emergence of RNA on the ancient Earth. Sophisticated internal control of lifetime is an indication of selection - of backbone structure, base modifications, and sequence, all of which modulate Goldilocks landscapes.

## DATA AVAILABILITY

All data are available through NAR online.

## Supplementary Material

gkad124_Supplemental_Files
